# Phase II study of weekly oxaliplatin plus infusional fluorouracil and folinic acid (FUFOX regimen) as first-line treatment in metastatic gastric cancer

**DOI:** 10.1038/sj.bjc.6602697

**Published:** 2005-07-12

**Authors:** F Lordick, S Lorenzen, J Stollfuss, U Vehling-Kaiser, F Kullmann, M Hentrich, R Zumschlinge, H Dietzfelbinger, J Thoedtmann, M Hennig, T Seroneit, R Bredenkamp, J Duyster, C Peschel

**Affiliations:** 1 Clinic Rechts der Isar, Technical University of Munich, Ismaninger Str. 22, 81675 Munich, Germany; 2 Oncological Outpatient Clinic, Heilig-Geist-Gasse 411, 84028 Landshut, Germany; 3 University Clinic of Regensburg, Franz-Josef-Strauss-Allee 11, 93053 Regensburg, Germany; 4 Clinic Harlaching, Sanatoriumsplatz 2, 81545 Munich, Germany; 5 Clinic Traunstein, Cuno-Niggl-Str. 3, 83278 Traunstein, Germany; 6 Schindlbeck-Clinic, Seestr. 43, 82211 Herrsching, Germany; 7 Sanofi-Aventis Group, Potsdamer Str. 8, 10785 Berlin, Germany; 8 Center for Clinical Studies, Ismaninger Str. 22, 81675 Munich, Germany

**Keywords:** oxaliplatin, fluorouracil, metastatic gastric cancer, first-line

## Abstract

Oxaliplatin plus fluorouracil/folinic acid (5-FU/FA) every 2 weeks has shown promising activity in advanced gastric cancer. This study assessed the efficacy and safety of weekly oxaliplatin plus 5-FU/FA (FUFOX regimen) in the metastatic setting. Patients with previously untreated metastatic gastric cancer received oxaliplatin (50 mg m^−2^) plus FA (500 mg m^−2^, 2-h infusion) followed by 5-FU (2000 mg m^−2^, 24-h infusion) given on days 1, 8, 15 and 22 of a 5-week cycle. The primary end point of this multicentre phase II study was the response rate according to RECIST criteria. A total of 48 patients were enrolled. Median age was 62 years and all patients had metastatic disease, with a median number of three involved organs. The most common treatment-related grade 3/4 adverse events were diarrhoea (17%), deep vein thrombosis (15%), neutropenia (8%), nausea (6%), febrile neutropenia (4%), fatigue (4%), anaemia (4%), tumour bleeding (4%), emesis (2%), cardiac ischaemia (2%) and pneumonia (2%). Grade 1/2 sensory neuropathy occurred in 67% of patients but there were no episodes of grade 3 neuropathy. Intent-to-treat analysis showed a response rate of 54% (95% CI, 39–69%), including two complete responses. At a median follow-up of 18.1 months (range 11.2–26.2 months), median survival is 11.4 months (95% CI, 8.0–14.9 months) and the median time to progression is 6.5 months (95% CI, 3.9–9.2 months). The weekly FUFOX regimen is well tolerated and shows notable activity as first-line treatment in metastatic gastric cancer.

Systemic chemotherapy has been shown to prolong survival and to relieve symptoms in advanced gastric cancer (Glimelius *et al*, 1997). However, the number of patients who benefit from treatment is still limited and the optimal chemotherapy regimen has not yet been defined. Platinum-free regimens exhibited disappointing efficacy in randomised trials. Cisplatin-based regimens, for example the combination of epirubicin, cisplatin, and 5-FU (ECF), have demonstrated superior efficacy, albeit with significant toxicity and inconvenience from the patients' perspective (Waters *et al*, 1999; Vanhoefer *et al*, 2000).

Oxaliplatin is a third-generation diaminocyclohexane platinum compound proven in numerous clinical trials to be active in various tumour types. In advanced colorectal cancer, treatment with oxaliplatin plus 5-fluorouracil/folinic acid (5-FU/FA) led to significantly increased overall survival and progression-free survival compared with former standard regimens (de Gramont *et al*, 2000; Giacchetti *et al*, 2000; Goldberg *et al*, 2004). Therefore, in many countries this combination forms the new standard of care in the first-line treatment of metastatic disease. In advanced gastric cancer, biweekly oxaliplatin plus 5-FU/FA has shown considerable antitumour activity in phase II trials (Louvet *et al*, 2002a; Al-Batran *et al*, 2004). Oxaliplatin shows a better toxicity profile than cisplatin. Its main and dose-limiting toxicity is acute, cumulative peripheral sensory neurotoxicity, resulting in acral paresthaesia and dysesthaesia, which are exacerbated by cold. The weekly application of oxaliplatin plus infusional 5-FU/FA (FUFOX), as proposed by the Arbeitsgemeinschaft Internistische Onkologie (AIO), has proven to be highly effective in first-line metastatic colorectal cancer. The AIO FUFOX regimen is also associated with an acceptable toxicity profile, with a particularly low rate of sensory neuropathy (Grothey *et al*, 2002).

This phase II trial was designed to assess the efficacy and safety of the weekly combination of oxaliplatin plus infusional 5-FU/FA (FUFOX) in first-line metastatic gastric cancer.

## METHODS

### Patient population

To be eligible for this study, patients had to have histologically confirmed metastatic adenocarcinoma of the stomach or oesophagogastric junction. At least one measurable lesion previously untreated with radiotherapy had to be present. No prior treatment for advanced disease was allowed. Patients were allowed to have received prior adjuvant or neoadjuvant chemotherapy/chemoradiation, providing that treatment had been completed ⩾6 months before inclusion in the study. All patients were >18 years of age with an ECOG performance status ⩽2 and a life expectancy of ⩾12 weeks.

Patients were required to have adequate bone marrow, renal and hepatic function, defined as an absolute neutrophil count (ANC) ⩾1500 *μ*l, platelets ⩾100 000 *μ*l, *S*-creatinine<1.5 × upper limit of normal (ULN), total bilirubin⩽1.5 × ULN, SGOT and/or SGPT⩽1.5 × ULN (⩽5.0 × ULN in the presence of liver metastasis). Patients with any central nervous system metastases or neuropathy ⩾grade 2 were excluded.

Pretreatment evaluation included signed written informed consent, complete history and physical examination, laboratory tests, CT scans of all areas affected by the tumour and an ECG. A urine or serum pregnancy test was performed in female patients of child-bearing age. All patients gave written informed consent before enrolment and the study was approved by the ethics committee for human research at the Technical University of Munich. The study conformed to the principles of the Declaration of Helsinki and its subsequent amendments.

### Treatment plan

Oxaliplatin (50 mg m^−2^) was administered simultaneously with FA (500 mg m^−2^) as a 2-h intravenous infusion, followed immediately by 5-FU (2000 mg m^−2^) as a continuous infusion over 24 h. Treatment was repeated every week. One cycle of treatment was defined as 4 weeks of treatment followed by 1 week of rest. In order to avoid severe neurotoxicity, administration of oxaliplatin was limited to every other week in patients receiving more than four cycles. Patients were treated until best response or until there was evidence of disease progression. Patients going off study were allowed to receive any second-line treatment as determined appropriate by their oncologist.

### Study evaluations

A baseline CT was performed within 2 weeks prior to study inclusion. After every second cycle of therapy, patients underwent follow-up CT scans for assessment of response according to RECIST criteria (Therasse *et al*, 2000). In cases where treatment was discontinued before tumour progression, CT scans were repeated every 3 months. Responses were to be confirmed within 4 weeks and were reviewed centrally by one independent radiologist (J Stollfuss). Patients were considered evaluable for response if they had received at least two cycles (10 weeks) of treatment, with at least one follow-up tumour assessment. Nonevaluable patients were included into the intention-to-treat analyses but reported as being not evaluable.

Patients were monitored every week for laboratory parameters and adverse events. With the exception of peripheral sensory neuropathy, all adverse events were graded using the National Cancer Institute Common Toxicity Criteria (NCI-CTC, version 2.0). Peripheral sensory neuropathy was graded according to a modified oxaliplatin-specific scale (Caussanel *et al*, 1990): grade 1 – paresthaesias/dysesthaesias of short duration with complete recovery before the next cycle; grade 2 – paresthaesias/dysesthaesias persisting between two cycles without functional impairment; grade 3 – permanent paresthaesias/dysesthaesias resulting in functional impairment. Patients going off study for reasons other than disease progression were evaluated every 3 months during follow-up visits.

### Statistical considerations

The primary end point of this investigator-initiated, multicentre, nonrandomised, open-label, phase II study was to determine the proportion of patients responding to weekly FUFOX. The required number of patients for this trial was calculated according to the Simon two-stage design (Machin and Campbell, 1997), assuming a minimal response rate (*π*_0_) of 30% and a worthwhile-to-detect-response-rate (*π*_1_) of at least 50%. With a power of 80% and a significance level of 5% for testing the hypothesis H_0_: *π*≤*π*_0_ *vs* H_1_: *π*⩾*π*_1,_ this resulted in a sample size of 15 for the first stage. If more than five out of 15 were observed, another 31 patients were to be recruited in the second stage. The drug combination was to be rejected if less than 19 out of 46 patients were observed.

All eligible patients were included in the response, safety and survival analyses. Time to progression (TTP: time from study entry until documented tumour progression) and overall survival (OS: time from study entry until death) were analysed according to the Kaplan–Meier method, and were updated to 15 October 2004. Statistical computations were performed using SPSS (version 12.0).

## RESULTS

### Patient characteristics

A total of 48 patients were enrolled at nine study sites between August 2002 and November 2003. Patient baseline characteristics are listed in Table 1. All patients had metastatic disease, with the liver, lymph nodes, peritoneum and lung being the predominant sites of metastases. The median number of involved organs was three (range 0–5). In total, 37 (77%) patients had newly diagnosed primary metastatic disease. In all of those patients, the primary tumour had been left *in situ* whereas 11 (23%) patients had recurrent disease after previous surgery intending to cure the primary.

### Feasibility and safety

The median number of completed cycles was 4 (range 0–6 cycles). Adherence to the planned doses of both oxaliplatin and 5-FU was high. Dose reductions of one or both agents to <80% of initial doses were required in <10% of patients. The median administered cumulative dose of oxaliplatin was 800 mg m^−2^.

All grade 1/2 treatment-related adverse events observed in more than 5% of patients are listed in Table 2. While it affected 67% of patients, sensory neuropathy was generally mild. All grade 3/4 treatment-related adverse events are listed in Table 2. The most common grade 3/4 events (affecting >5% of patients) were diarrhoea, deep vein thrombosis, neutropenia and nausea. Of note, stomatitis and sensory neuropathy >grade 2 were not reported in any of the patients.

Serious adverse events (SAEs) were reported in 14 patients. Two SAEs, one case of septic diarrhoea with neutropenia and one of stroke were fatal. Both deaths occurred during the first two treatment cycles. As there were no other early deaths within the first 2 months after study entry, the 60-day mortality rate was 4.2%.

### Efficacy

Tumour response was evaluable according to RECIST criteria in 45 patients. Three (6%) patients who did not undergo the first follow-up tumour assessment due to early death or premature termination of therapy (patient preference) were included in the intention-to-treat analyses as nonevaluable subjects. There were two (4%) complete responses and 24 (50%) patients achieved a partial remission, resulting in an overall response rate of 54% (95% CI, 39–69%). Eight (17%) patients had stable disease and 11 (23%) had progressive disease at the first follow-up tumour assessment.

A total of 25 (52%) patients received second- and third-line chemotherapies: 13 irinotecan-based, seven taxane-based, three platinum-based, one epirubicin, one etoposide, and four investigational drugs. Two patients underwent secondary palliative surgery and one patient was treated with external beam radiation therapy.

**Figure 1:**
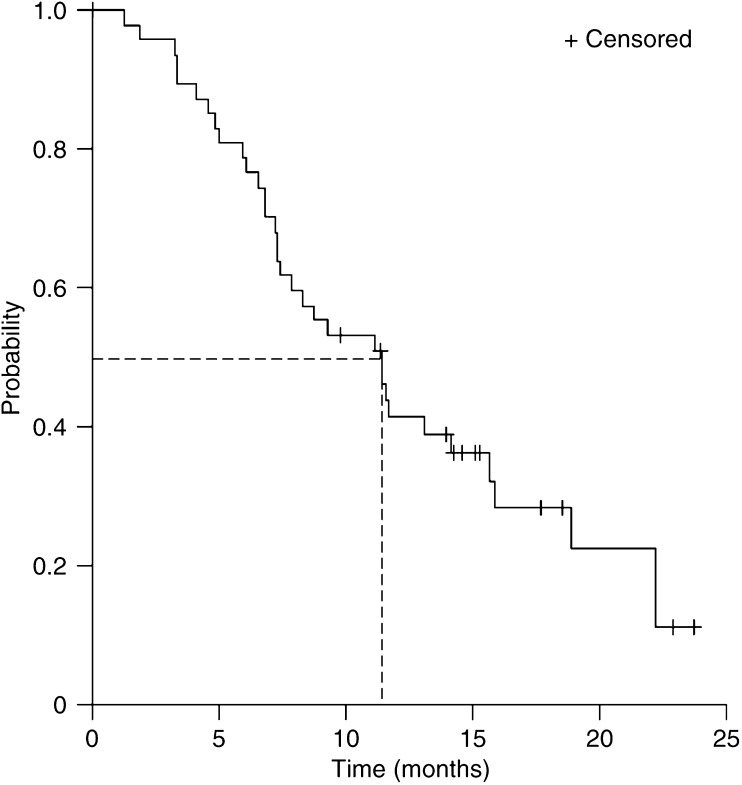
Kaplan–Meier curve for overall survival in all patients (*n*=48).

**Figure 2:**
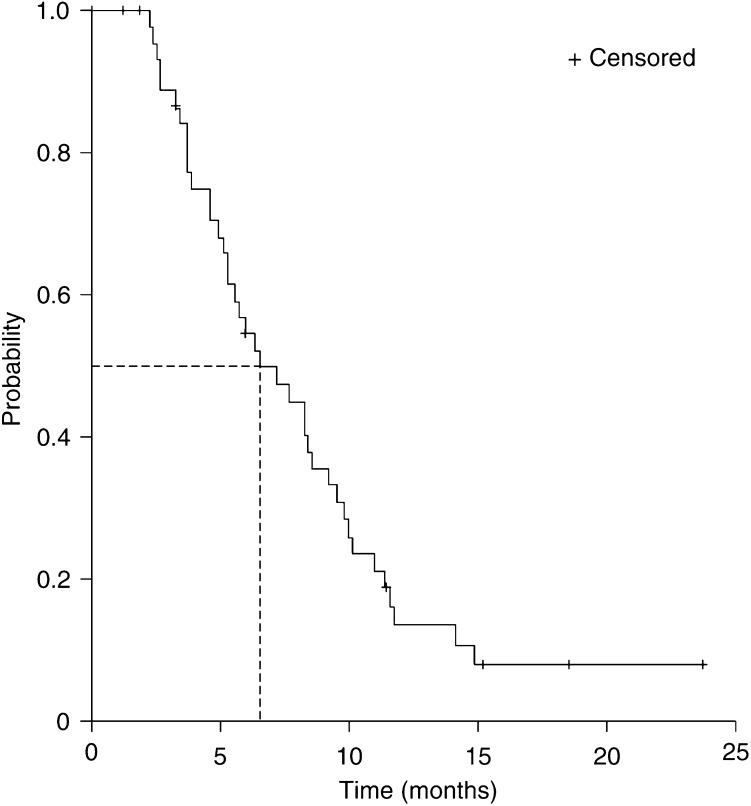
Kaplan–Meier curve for time to progression in all patients (*n*=48).

Median OS was 11.4 months (95% CI, 8.0–14.9 months, Figure 1). Median TTP was 6.5 months (95% CI, 3.9–9.2 months, Figure 2). Median follow-up is 18.1 months (range 11.2–26.2 months).

## DISCUSSION

Oxaliplatin combinations have become the mainstay of systemic treatment for advanced gastrointestinal tumours. As a result of the data from randomised phase III trials showing superiority over former standard regimens (de Gramont *et al*, 2000; Giacchetti *et al*, 2000; André *et al*, 2004a; Goldberg *et al*, 2004), oxaliplatin plus 5-FU/FA has been approved in many countries for the palliative treatment of stage IV colorectal cancer and the adjuvant therapy of stage III colon cancer. Moreover, there is increasing evidence from phase II studies that oxaliplatin combinations have significant activity in other gastrointestinal tumours such as pancreatic cancer (Louvet *et al*, 2002b; Ducreux *et al*, 2004) and biliary tract adenocarcinoma (André *et al*, 2004b). In combination with radiation therapy, oxaliplatin and fluoropyrimidines result in promising tumour remissions in locally advanced oesophageal and rectal cancer (Freyer *et al*, 2001; Khushalani *et al*, 2002; Rödel *et al*, 2003).

In advanced gastric cancer, two previously published phase II studies have shown consistent results regarding the activity of biweekly oxaliplatin-5–FU/FA combinations. These regimens induced objective tumour responses in 43 and 45% of patients, respectively, and were associated with a median survival of 9.6 and 8.6 months, respectively (Louvet *et al*, 2002a; Al-Batran *et al*, 2004). A study from Taiwan assessed the activity of oxaliplatin-5-FU/FA given on days 1 and 8, repeated every 3 weeks. This regimen induced an objective tumour reponse in 56% of the evaluable patients. The median TTP and survival were 5.2 and 10.0 months, respectively (Chao *et al*, 2004). Although data from phase III trials have not yet been presented, in daily practice the combination of oxaliplatin plus 5-FU/FA is increasingly used by many oncologists for the treatment of advanced gastric cancer.

The objective of this study was to assess the efficacy of weekly oxaliplatin plus 5-FU/FA given 4 out of 5 weeks (FUFOX regimen) as first-line treatment for metastatic gastric and oesophagogastric adenocarcinoma. Leading to an objective response rate of 54%, the activity of FUFOX exceeded the expectations of the trial. This unexpectedly high activity of FUFOX seems to translate into a meaningful clinical benefit, as indicated by the median survival time of 11.4 months.

One might assume that these promising efficacy results are attributable to a patient selection bias. To encounter this well-known phenomenon in phase II testing, selected study centres included two university hospitals, two community hospitals and five private practices. This represents the typical treatment facilities for cancer patients in Germany. As in three studies on oxaliplatin plus 5-FU/FA in gastric cancer previously published by the French group (Louvet *et al*, 2002a), the Taiwan group (Chao *et al*, 2004) and the Frankfurt group (Al-Batran *et al*, 2004), the majority of patients in our study presented with a good performance status and the median age of 62 years was in the same range as in the three previous trials. Of note, all patients included in our study presented with metastatic disease. Consequently, none of them underwent secondary curative treatment. In contrast, the French group reported on seven of 54 (13%) patients with locally advanced disease and on eight (15%) patients undergoing complementary surgery or chemoradiation with curative intent.

The three other mentioned studies do not report on the frequency of second-line chemotherapy in their study populations. In our study, the use of second- and even third-line therapies was relatively frequent (>50% of patients). The chosen regimens most frequently included irinotecan or taxanes. Although no phase III trial has yet defined the clinical benefit of second-line chemotherapy in gastric cancer, there is no doubt that second-line treatment with the most recently available agents may be effective in a considerable number of patients. Evidence from phase II trials suggests that irinotecan-containing regimens may be particularly active in this setting (Ajani *et al*, 2002; Assersohn *et al*, 2004). Therefore, it is important to acknowledge that the use of second-line chemotherapies may have positively influenced the survival time in our study population.

Treatment compliance for the FUFOX regimen was very good with a dose adherence to both oxaliplatin and 5-FU of >90%. As already observed by the Taiwan and even more by the Frankfurt group, avoiding the 5-FU bolus administration has led to a dramatic reduction of haematological toxicity. In the French regimen, which included a 5-FU bolus on day 1, the rate of grade 3/4 neutropenia was 38%. This was associated with an 11% rate of febrile neutropenia. Thus, in our study using weekly oxaliplatin/5-FU as well as in the modified version of biweekly FOLFOX as used by the Frankfurt group, bolus 5-FU was omitted. Consequently, the rate of grade 3/4 neutropenia was below 10% and febrile neutropenia occurred in less than 5% of the patients in both studies. Compared with the other oxaliplatin/5-FU regimens studied in gastric cancer, the rate of severe diarrhoea was relatively high (17%) in our study. However, this is comparable with recently reported findings with the FUFOX regimen in metastatic colorectal cancer, where grade 3/4 diarrhoea affected 21% of patients (Grothey *et al*, 2002). It would be worthwhile to investigate whether administering a lower dose of FA, which can itself cause diarrhoea, would reduce this rate.

Unfortunately, in our study, one patient died as a result of septic diarrhoea and neutropenia. The investigators considered this death to be treatment-related. However, the relationship between stroke and death in another patient was judged to be uncertain by the investigators. Nevertheless, these events highlight the importance of careful monitoring of these highly vulnerable patients, even when a generally well-tolerated chemotherapy regimen is administered.

As expected with oxaliplatin-containing regimens, neurotoxicity affected the majority of patients. However, in contrast to studies with the FOLFOX-6 regimen, where 21% of patients reported functional impairment caused by sensory neuropathy with a median administered oxaliplatin dose of 900 mg m^−2^ (Louvet *et al*, 2002a), the intensity of neuropathy observed in our trial, where the median dose of oxaliplatin was 800 mg m^−2^, did not exceed mild to moderate grades. This observation corresponds well with the recently reported low rate of severe neuropathy with the weekly FUFOX regimen in stage IV colorectal cancer (Grothey *et al*, 2002). These observations indicate that weekly application of lower doses of oxaliplatin may have advantages compared with biweekly oxaliplatin-regimens in terms of neurotoxicity.

In conclusion, weekly oxaliplatin plus 5-FU/FA has a favourable safety profile compared with previous data from studies of the biweekly FUFOX regimen. This weekly FUFOX regimen results in a high tumour response rate in first-line metastatic gastric cancer and is associated with a promising OS time. Therefore, the weekly FUFOX regimen can be recommended for phase III studies as well as a combination partner for new investigational drugs in phase I/II trials.

## Figures and Tables

**Table 1:** Patient baseline characteristics

**Characteristics**	
Total number of patients	48
Median age (range)	62 (41–75) years
	
*Gender*	
Male	39 (81%)
Female	9 (19%)
	
*ECOG performance status*	
0	23 (48%)
1	24 (50%)
2	1 (2%)
	
*Disease status*	
Newly diagnosed	37 (77%)
Recurrent	11 (23%)
Locally advanced	0 (0%)
Metastatic	48 (100%)
	
*Localisation of the primary tumour*	
Stomach	23 (48%)
Oesophagogastric junction	23 (48%)
Gastric stump	2 (4%)
	
*Sites of disease*	
Liver	30 (63%)
Lymph nodes	29 (60%)
Peritoneum	16 (33%)
Lung	13 (27%)
Bone	5 (10%)
Others^a^	6 (12%)

ECOG=Eastern Cooperative Oncology Group.

a Skin (two patients), scrotum, adrenals, spleen and kidney.

**Table 2:** Treatment-related adverse events

	**No. of patients (%)**	
**Event**	**Grade 1/2**	**Grade 3/4**
Anaemia	21 (44%)	2 (4%)
Neutropenia	6 (13%)	4 (8%)
Febrile neutropenia	NA	2 (4%)
Thrombocytopenia	7 (15%)	1 (2%)
Diarrhoea	17 (35%)	8 (17%)
Constipation	4 (8%)	0 (0%)
Stomatitis	7 (15%)	0 (0%)
Nausea	33 (69%)	3 (6%)
Emesis	13 (27%)	1 (2%)
Fatigue	21 (44%)	2 (4%)
Deep vein thrombosis	NA	7 (15%)
Tumour bleeding	NA	2 (4%)
Cardiac ischaemia	NA	1 (2%)
Pneumonia	NA	1 (2%)
Fever	4 (8%)	0 (0%)
Alopecia	7 (15%)	NA
Sensory neuropathy	32 (67%)	0 (0%)

NA=not applicable.
